# An In-Vitro Comparative Evaluation of Pre-treatment With Nano-Silver Fluoride on Inhibiting Secondary Caries at Tooth Restoration Interface

**DOI:** 10.7759/cureus.7934

**Published:** 2020-05-02

**Authors:** Konica J Nanda, Shilpa Naik

**Affiliations:** 1 Department of Pedodontics and Preventive Dentistry, D.Y. Patil University School of Dentistry, Mumbai, IND

**Keywords:** nano-silver fluoride, secondary caries, restoration, glass ionomer cement, composite resin, artificial caries, microhardness

## Abstract

Introduction and aim

Dental caries is the global burden worldwide and has a negative effect on the quality of life. Restorative materials in pediatric dentistry have shown satisfactory properties; however, the highest failures are still reported due to the occurrence of secondary caries. The article aims to assess the effectiveness of pre-treatment with nano-silver fluoride (NSF) in inhibiting secondary caries at the tooth restoration interface.

Materials and methods

Forty tooth specimens were prepared from 20 newly extracted human premolar teeth by sectioning the tooth mesiodistally. Class V cavities were prepared on each specimen at enamel dentin junction. Specimens were randomly distributed into four groups (n=10): 1) glass ionomer cement (GIC) restoration, 2) composite restoration 3) NSF pre-treatment + GIC restoration, 4) NSF pre-treatment + composite restoration. After sterilization, specimens were subjected to artificial caries formation by pH cycling method for 14 days. Specimens were sectioned and mounted to evaluate the demineralization by using the Vickers microhardness test. Outer lesion depth was measured at the tooth restoration interface on digital radiographs. Data was analyzed using Student unpaired t-test, one-way ANOVA, and Tukey honestly significant difference (HSD) post hoc test.

Results

The mean microhardness value of pre-treated GIC and composite group with NSF was more than the non-treated NSF group, with a significant difference at the enamel, indicating lesser demineralization. Outer lesion depth was lesser in the pre-treated group showing better tooth restoration integrity with a statistically significant difference between the groups.

Conclusion

Pre-treatment with NSF is beneficial in increasing the resistance of GIC and composite resin restoration to secondary caries formation.

## Introduction

Scientific research has provided overwhelming evidence that restoration failure was mainly attributed to secondary caries in both primary as well as permanent dentition [1, 2]. The Federation Dentaire International (FDI) has defined secondary caries as a “positively diagnosed carious lesion, which occurs at the margin of existing restoration” [3]. Secondary caries is found in two dimensions: one on the tooth surface and other on the cavity wall. Fluoride has potent properties of inhibiting demineralization and enhancing remineralization. Restorative materials with fluoride ion release, including the pre-treatment solutions are found to be equally effective in reducing secondary caries [4].

Systematic review and meta-analysis on silver diamine fluoride (SDF) have proved its ability to arrest carious progression and to prevent the occurrence of a new carious lesion in children [5, 6]. SDF, when applied on the tooth surface, leads to bacterial killing, thus preventing biofilm formation. It leads to the conversion of hydroxyapatite crystals to fluorapatite crystals, which are more resistant to demineralization. However, SDF has a known adverse effect of staining the carious tissue black due to oxidation of the ionic silver particles [7]. A new experimental formulation, nano-silver fluoride (NSF), was prepared using silver nanoparticles, chitosan, and fluoride. It has both preventive and antimicrobial properties like SDF and proved to be an effective anti-caries agent [8]. However, the staining of porous dental tissues, as seen with SDF and amalgam, was eliminated due to the nanoparticle size of silver [9].

A literature search in Pubmed did not reveal studies in English on pre-treatment with NSF to prevent secondary caries. Hence the present study aims to assess the effectiveness of pre-treatment with nano-silver fluoride in inhibiting secondary caries at the tooth restoration interface. The null hypothesis tested was that pre-treatment with nano-silver fluoride has no effect on secondary caries prevention in glass ionomer cement and composite resin restoration.

## Materials and methods

Experimental design

**Figure 1:**
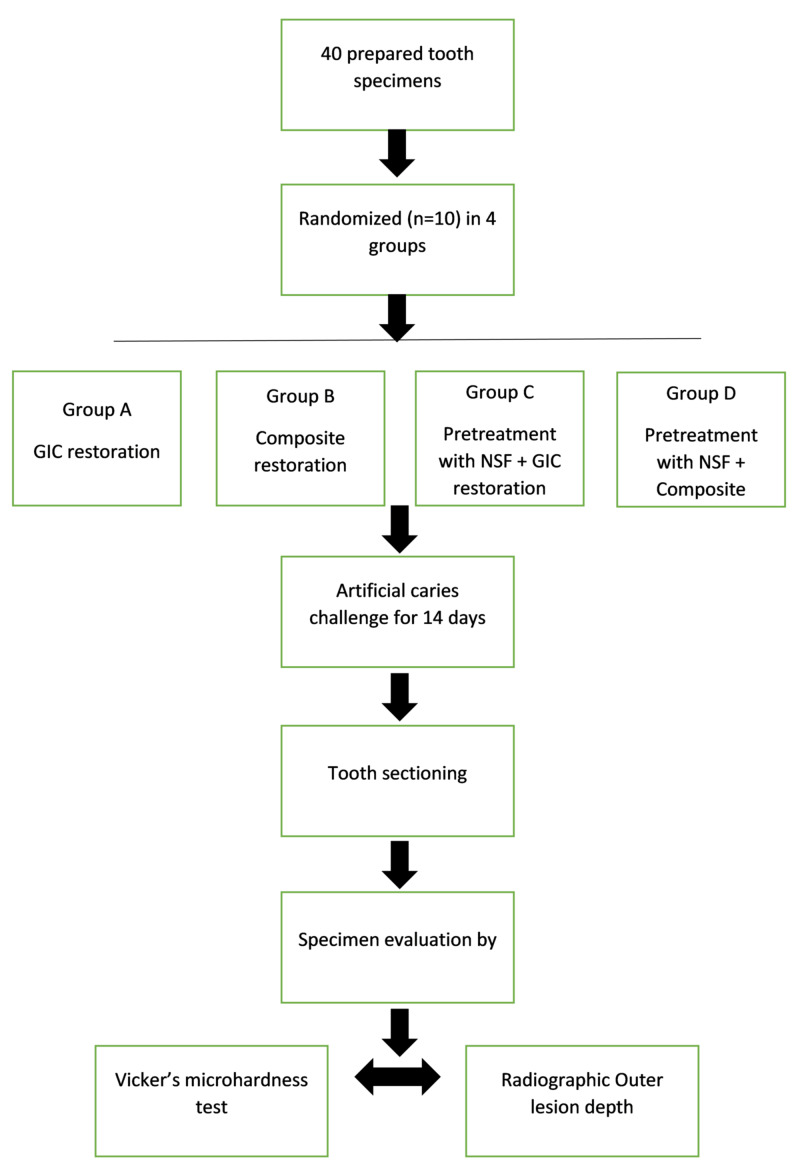
Experimental design GIC - glass ionomer cement; NSF - nano-silver fluoride

The study design was a randomized, controlled, single-blind (at the investigator level) laboratory study. After the approval by the Institutional Ethical Committee (reference number FRC/2018/Pedo/24), 40 specimens were randomly separated into four groups depending on the different treatment applications of pre-treatment with nano-silver fluoride or no application and followed by glass ionomer cement (GIC) or composite resin restoration. Specimens were subjected to artificial caries challenge for 14 days and were evaluated by Vickers microhardness test and radiographic outer lesion depth. The experimental design is illustrated in Figure 1.

Preparation of nano-silver fluoride

Colloidal silver was prepared by dissolving 1.0g of chitosan in 200 mL of acetic acid 2% (V/V). The solution was stirred overnight and filtered under vacuum. Subsequently, an aliquot of 60 mL of chitosan solution was placed in an ice bath while being stirred, and 4.0 mL was added to a solution of silver nitrate 0.012 mol L-1, 30 min prior to the addition of sodium borohydride. The relationship between AgNO3 and NaBH4 was maintained at a 1:6 mass and added dropwise. The reduction of Ag+ was initiated immediately, as the solution changed from colorless to light yellow and ended up reddish. The silver nanoparticles had an average size of 3.2 ± 1.2 nm and a spherical shape. Fluoride (NaF) was added at the end of the experiment. The concentrations of each component, as expressed in micrograms per millilitre, were as follows: chitosan (28,585 mg/mL); Ag+ (376.5 mg/mL) and sodium fluoride (5028.3 mg/mL) [9].

Preparation of the specimen

Twenty human premolar teeth newly extracted for the orthodontic purpose were selected. Teeth were checked to be free of caries, malformation, and cracks with the magnifying glass. Later, they were stored in a 0.1% aqueous thymol solution. Teeth were subjected to debridement by periodontal scalpel and curette. The fragments were sterilized in an autoclave (one atmospheric pressure - 121 °C for 20 minutes) to ensure the absence of any type of microorganism that could interfere in the results and to avoid contamination [10].

Teeth were longitudinally sectioned into two halves - buccal and lingual obtaining 40 specimens. Class five cavity was prepared on each specimen (3×2×1.5mm) with a standardized diamond flat cylindrical bur (SF-21, MANI Inc, Japan) with occlusal margin in the enamel and cervical margin in the dentin. Burs were changed after every ten-cavity preparations so the uniformity of the preparation was maintained. Restorative material used as mentioned in Table 1. 

**Table 1:** Used materials

Material	Manufacturer
Glass ionomer cement	Ketac™-Molar (3M ESPE, St Paul, USA)
Composite resin	Filtek™​​​​​​​ Z250 (3M ESPE, St Paul, USA).
Bonding agent	Single Bond Universal (3M ESPE, St Paul, USA)

Forty specimens were randomly divided into four groups depending on the treatment application as follows:

**Group 1: **the cavity was bulk filled with glass ionomer cement.

**Group 2:** the cavity was treated with a single step bonding agent for 20 seconds, air-dried and light-cured for 15 seconds, subsequently composite was filled with the layering technique.

**Group 3:** the cavity was conditioned with NSF for two minutes followed by GIC restoration.

**Group 4: **the cavity was conditioned with NSF for two minutes followed by a single step bonding agent and composite filled with the layering technique.

The restorations were polished using aluminum oxide discs of medium and fine granulation (Sof-Lex™, 3M, Saint Paul, USA). Acid-resistant varnish was applied to the specimen leaving one millimeter from the tooth restoration interface.

Artificial caries induction by pH cycling

The artificial carious lesion was created from previously described methods [11-13]. The demineralizing solution contained 2.2 mM CaCl2, 2.2 mM NaH2PO4, and 50 mM acetic acid adjusted to a pH of 4.8. The remineralizing solution contained 1.5 mM CaCl2, 0.9 mM NaH2PO4, and 0.15 M KCl adjusted to a pH of 7.0. Each specimen was cycled in 10 ml of both solutions for eight hours in the demineralizing solution and 16 hours in the remineralizing solution. This procedure was carried out for 14 days at room temperature without agitation.

Hardness evaluation

Teeth were sectioned perpendicular to the long axis in two halves under cooling. One half was mounted in epoxy resin so that the area to be analyzed remained exposed. The surfaces for the microhardness tests were polished using 600 and 1200 grit aluminum oxide abrasive paper. A microhardness test was done to evaluate demineralization around the restoration. Vicker’s microhardness indenter was used to perform indent on the surfaces at the depth of 25 µm and at a distance of 50 µm from the tooth restoration interface in enamel and dentin at a load of 980.7 mN for an interval of 10 seconds.

Radiographic evaluation of outer lesion depth

**Figure 2:**
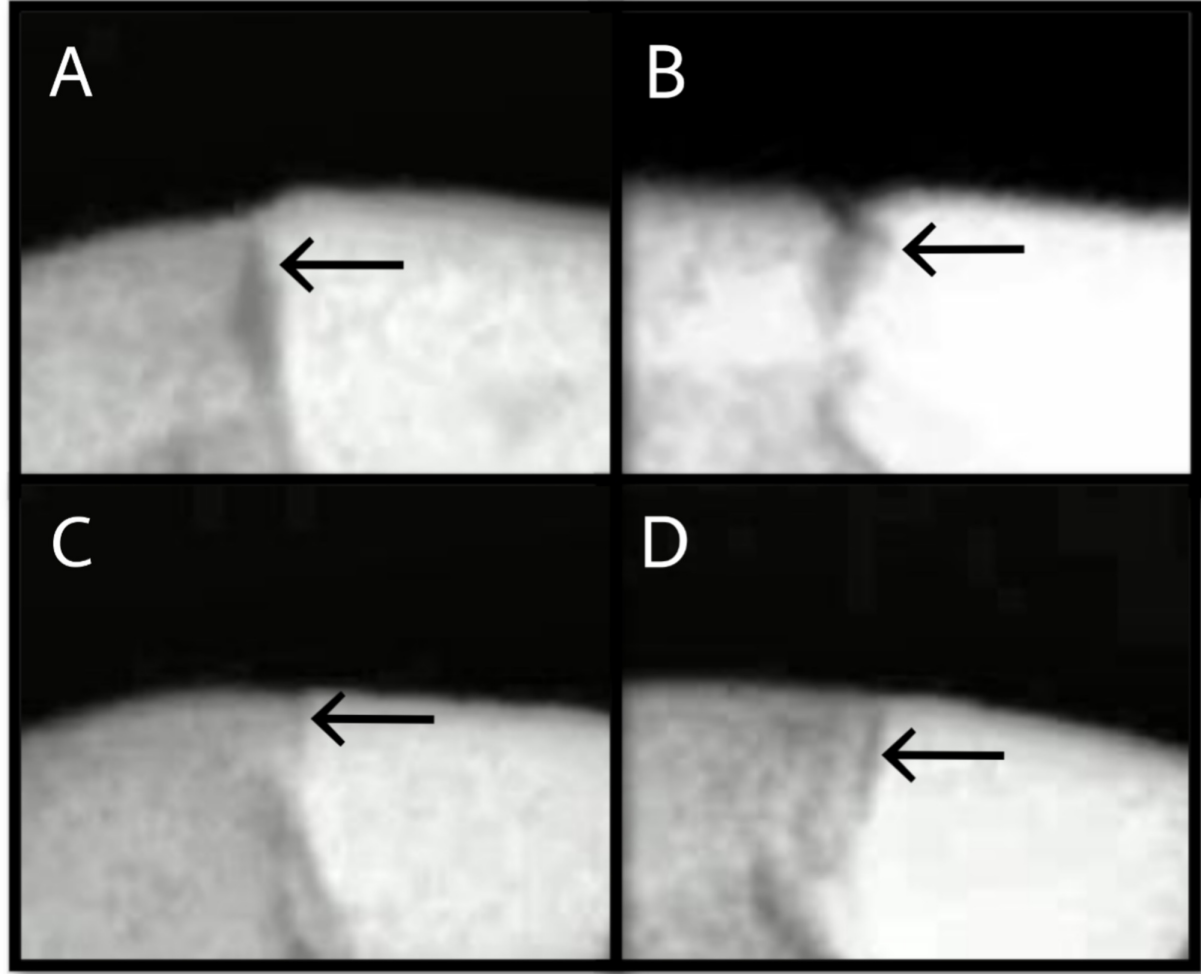
Representative digital radiography images of outer lesion depth, according to the groups A: GIC restoration; B: composite restoration; C: NSF + GIC restoration; D: NSF + composite restoration. All images present a demineralized area on the outer surface at the tooth restoration interface. GIC - glass ionomer cement; NSF - nano-silver fluoride

Teeth were scanned using X-ray digital radiography on Dental Imaging Software version 6.14.0 (Carestream Health Inc, New York, US) to assess outer lesion depth. The X-ray source was operated at a voltage of 60kV and a current of 7mA. The method of lesion assessment on the restoration-tooth interface was adapted from Hsu et al. by assessing the outer lesion depth (the deepest point of the lesion from the tooth surface) [14]. The start and endpoints of the outer lesion were determined according to the corresponding grey value. Representative images of digital radiography according to the group are seen in Figure 2. 

Statistical analysis

Descriptive and inferential statistical analyses were carried out in the present study. Results on continuous measurements were presented as mean ± standard deviation (SD). Level of significance was set at p=0.05 and any value less than or equal to 0.05 was considered to be statistically significant. Microhardness and radiographic outer lesion depth values were compared between groups with Student t-tests (two-tailed, unpaired), one-way analysis of variance (ANOVA), and Tukey honestly significant difference (HSD) post hoc test. The statistical software IBM SPSS statistics 20.0 (IBM Corp., Armonk, US) was used for the analyses of the data.

## Results

The group receiving NSF pre-treatment presented higher hardness means at a depth of 25µm from the outer tooth surface in both enamel and dentin regardless of the restoration used as seen in Table 2. There was a statistically significant difference observed when microhardness at enamel was compared between the groups (p=0.008). Further using Tukey’s post hoc analysis, a significant difference was observed between GIC and NSF + GIC (p=0.021) and between composite and NSF + GIC (p=0.009). However, no statistically significant difference was observed when microhardness at dentin was compared. 

**Table 2:** Comparison of the microhardness at enamel and dentin with different materials using ANOVA test ^a,b ^Same lower-case alphabets indicate a significant difference (one-way ANOVA and Tukey post hoc test p<0.05). GIC - glass ionomer cement; NSF - nano-silver fluoride; SD - standard deviation; ANOVA - analysis of variance

Group	n	Mean hardness at enamel (SD)	Mean hardness at dentin (SD)
GIC	10	230.20 (32.97)^a^	67.66 (11.38)
Composite	10	223.20 (17.96)^b^	58.86 (7.14)
NSF + GIC	10	287.00 (34.964)^ab^	79.34 (16.59)
NSF + composite	10	246.20 (17.82)	70.04 (6.83)
Total	40	246.65 (35.57)	68.97 (12.72)

Table 3 shows the result for radiographic outer lesion depth measurement after the artificial caries challenge. Lowest score was observed in the pre-treated group on comparison with the control group indicating lesser demineralization of the pre-treated group and better tooth integrity. There was a statistically significant difference observed when groups were compared (p=0.029). Further using Tukey’s post hoc analysis significant difference was observed between composite and NSF + GIC (p=0.033) and between composite and NSF + composite (p=0.05). 

**Table 3:** Comparison of the radiographic outer lesion depth at tooth restoration interface with different materials ^a,b ^Same lower-case alphabets indicate a significant difference (one-way ANOVA and Tukey post hoc test p<0.05). GIC - glass ionomer cement; NSF - nano-silver fluoride

Group	GIC	Composite	NSF + GIC	NSF + composite
n	10	10	10	10
Mean outer lesion depth	0.280 (0.08)	0.420 (0.19)^ab ^	0.180 (0.10)^a^	0.200 (0.07)^b^

## Discussion

The purpose of the study was to examine if pre-treatment with nano-silver fluoride could prevent secondary caries formation. Based on the results of the present study, null hypothesis was rejected. NSF could thus be recommended as a pre-treatment solution prior to any restoration for prevention of the secondary caries.

Glass ionomer cement having the property of chemical adhesion to the tooth surface and fluoride release is the most chosen restorative material in pediatric dentistry. However, it has been reported in a study by Wiegand et al. that fluoride release rapidly decreases from the glass ionomer cement in one to three days and stays constant thereafter [15]. Also, lower fracture strength and wear resistance are known disadvantages.

Conversely, composite resin restorations are highly esthetic and simulate the appearance of the natural tooth. However, it is a highly technique sensitive procedure that is seldom substituted by glass ionomer cement restoration because of its single increment technique, favoring better management [16]. Hence, glass ionomer cement and composite resin is the most accepted restoration in pediatric dentistry, was chosen as the material of choice.

Studies have been reported on the antibacterial efficacy of silver nanoparticles against Streptococcus mutans [17]. Nano-silver particles being smaller in size and spherical have larger contact surfaces increasing the antimicrobial activity. These particles do not form oxides when contacting oxygen in the medium, preventing the darkening of the demineralized enamel [9]. Thus, nano-silver particles are powerful antimicrobial agent which prevents unesthetic changes at a low production cost [18]. Fluoride is a known antibacterial agent against Streptococcus mutans [19]. Chitosan was used to stabilize silver nanoparticles as described by Wei et al. and is toxic to several bacteria, fungi, and parasites [20, 21]. In a study by Scarpelli et al., silver nanoparticles were able to inhibit 100% microorganism growth at a lower concentration than required for Saforide, Cariestop, and Ancarie (commercially available silver diamine fluoride [SDF] formulations). Also, Scarpelli et al. suggested adding fluoride to the solution because of its known benefits but however there was difficulty in stabilizing silver nanoparticles [22]. Research on this aspect of stabilizing silver nanoparticles, lead to a finding that chitosan was a better agent to stabilize silver nanoparticles. Synergistic action of nano-silver particles, fluoride and chitosan are responsible for the prevention of biofilm formation and subsequent demineralization of the tooth surface and secondary caries.

Bacterial adhesion plays an important role in biofilm formation and the pathogenesis of caries since glucan produced by bacterial glucosyltransferase enzymes promotes adhesion between cell-cell and cell-surfaces [23]. Silver ions show the ability to prevent biofilm formation by inhibiting this adhesion.

For artificial caries formation, pH cycling was chosen because of its ability to form caries substrate which is similar to the affected caries dental layer, after caries removal [13].

Hardness test was not done prior to the artificial caries challenge, to avoid the cracks formed on the surface of the specimen on testing. Outer lesion depth was used to evaluate the integrity of the tooth restoration interface. Also, restorative material plays an important role in the development of wall lesion. However, wall lesion is most commonly associated with the size of the gap between the tooth and the restoration interface. As it is less associated with the formation of secondary caries, this parameter was not evaluated in the present study.

Superficial microhardness has proved to be a reliable detector to check for the effectiveness of dental tissue remineralization. In the present study pre-treatment group had higher hardness value compared to the conventional restoration procedure. This indicates that demineralization around the restoration was lower in the pre-treatment group. Hence, pre-treatment with NSF prior to restoration gives a better prognosis for the prevention of secondary caries. Radiographic outer lesion depth did not show a statistically significant difference from the conventional restoration group. However, it had a better tooth restoration integrity than the conventional group.

In a prospective controlled clinical trial by Santos et al., 66.7% of the lesion treated with NSF was arrested at 12 months which was similar to the caries arrest rate with silver diamine fluoride [9]. Targino et al. evaluated the antimicrobial and cytotoxicity of nano-silver fluoride against Streptococcus mutans in comparison to chlorhexidine and silver diamine fluoride. NSF was found to be effective against S. mutans in much lower doses, may have lower toxicity than SDF, and not stain teeth [8].

Teixeria et al. studied experimental dentifrice containing NSF in comparison to sodium fluoride (NaF) dentifrice against S. mutans and found that NSF dentrifice showed a lower minimal inhibitory concentration with respect to preventing bacterial adhesion and pH decreases. NSF had a better antibacterial activity and potential effectiveness to prevent caries [24].

In the same way as SDF, NSF treatment is inexpensive and can be afforded by even the lower socio-economic group. Pre-treatment with this agent will prevent the chances of new caries formation with its antimicrobial and remineralizing property. Hence, can be a definitive type of treatment especially when considering mass treatment.

## Conclusions

NSF is beneficial in the prevention of secondary caries formation, solving the most common cause of failure of restorations. The available literature on NSF has displayed its efficacy in arresting caries, with antimicrobial properties analogous to SDF. It is low in cytotoxicity, economical, and importantly has the potential advantage of not staining the dental tissue black, overcoming the drawback of SDF. Thus, NSF can be a global boon in treating mass population, unco-operative patients and those with special health care needs. However, more comprehensive research with extensive in-vivo studies should be conducted to investigate further in this aspect.
